# Complication Rates are Low for Women Aged 70 and Older Undergoing Sacrocolpopexy

**DOI:** 10.1007/s00192-025-06277-w

**Published:** 2025-09-08

**Authors:** Tien C. Nguyen, Halina Zyczynski, Mary F. Ackenbom, Stephanie W. Zuo

**Affiliations:** 1 University of Pittsburgh School of Medicine, Pittsburgh, PA USA; 2 Division of Urogynecology and Reconstructive Pelvic Surgery, Department of Obstetrics and Gynecology, Magee-Womens Hospital, University of Pittsburgh Medical Center, Pittsburgh, PA USA; 3 Magee-Womens Research Institute, Pittsburgh, PA USA; 4 Division of Urogynecology and Reconstructive Pelvic Surgery, Department of Obstetrics and Gynecology, University of Virginia, Charlottesville, VA USA

**Keywords:** Sacrocolpopexy, Geriatrics, Aging, Adverse events

## Abstract

**Introduction and Hypothesis:**

Aging may place patients at greater risk for adverse perioperative outcomes. We hypothesized that women undergoing minimally invasive (MIS) sacrocolpopexy aged ≥ 70 years are more likely to experience adverse events (AE) within 8 weeks of surgery.

**Methods:**

We performed a secondary analysis of a retrospective study on perioperative adverse events in women ≥ 61 years old undergoing prolapse surgery at a single academic center from January 2016 to May 2023. Only women undergoing MIS sacrocolpopexy were included. The primary outcome was a composite 8-week AE outcome. Secondary outcomes included prolapse recurrence and mesh complication. Variables were compared between the groups using *t*-test (or Mann–Whitney U) and chi-square (or Fisher’s exact) analyses. Multivariable logistic regression was performed, controlling for variables with *p* < 0.05 on univariate analysis.

**Results:**

Of the 709 women who underwent MIS sacrocolpopexy, 29.9% (*n* = 212) were aged ≥ 70 years. Age ≥ 70 was not significantly associated with 8-week perioperative AEs, nor was it associated with prolapse recurrence or mesh complication. The composite AE outcome was not associated with age ≥ 70 on multivariable analysis, controlling for CCI score, robotic approach, and concomitant hysterectomy (adjusted odds ratio (aOR) 0.64, 95% confidence interval (CI) [0.38–1.10]). Women aged ≥ 70 years had a 73% lesser adjusted odds of surgical site infections on multivariable analysis (95% CI [0.08–0.93]).

**Conclusions:**

Age ≥ 70 years is not associated with perioperative AEs, prolapse recurrence, or mesh complication. These findings highlight the safety profile of this surgical approach in older women, an important consideration for urogynecologic surgeons caring for an aging population.

## Introduction

Aging is a risk factor for pelvic floor disorders, including pelvic organ prolapse where pelvic organs descend and bulge into the vagina [1]. As the older adult population in the United States increases, the number of women with prolapse is also expected to substantially increase [2]. Surgical management can improve the quality of life of patients with symptomatic prolapse who have either failed or declined more conservative measures like pelvic floor therapy or a pessary [3]. In the United States, the lifetime risk of undergoing surgery for prolapse is 12.6% [4]. Roughly 15% of women aged 65 or greater undergo surgical management for prolapse [5].

Sacrocolpopexy is a procedure that involves using mesh to suspend the apex of the vagina to the anterior longitudinal ligament on the sacral promontory. Nowadays, the procedure is typically done using minimally invasive (MIS) approaches, which include conventional laparoscopic or robotic techniques [6]. Studies have shown that MIS sacrocolpopexy is an effective procedure for the treatment of apical prolapse [7, 8]. Surgeons may avoid sacrocolpopexy in older patients because the procedure is complex and has the potential for complications. In particular, MIS approaches often employ Trendelenburg positioning and expose patients to CO_2_ during pneumoperitoneum to improve visualization of pelvic organs, both of which may compromise postoperative cognitive status [9–11]. Additionally, surgeons may consider the fact that aging is associated with multimorbidity and less resilience to surgical trauma. Some studies have established MIS sacrocolpopexy as a feasible option in older patients with complication rates comparable to younger patients [12, 13], while other studies show an increased risk of perioperative adverse events following MIS sacrocolpopexy in older women [14]. One study by Gluck et al. examined long-term complications of mesh exposure and prolapse recurrence after laparoscopic sacrohysteropexy and sacrocolpopexy and found no difference by age [15]. Despite varying results, few studies report complication rates beyond 30 days, and data on long-term follow-up of mesh complication and prolapse recurrence are limited [12–14, 16, 17]. We sought to expand on this area of research by examining a larger cohort of older patients undergoing MIS sacrocolpopexy specifically, as older, high-functioning patients and their surgeons may elect this gold-standard procedure for prolapse repair [18]. More robust data on the risks of sacrocolpopexy in older patients can provide urogynecologists with crucial information about its safety and efficacy.

Considering the elevated risks of surgery in older patients and some evidence of increased postoperative adverse events (AEs) among this population, we hypothesized that women undergoing MIS sacrocolpopexy aged ≥ 70 years are more likely to experience AEs within 8 weeks of surgery compared to their younger counterparts.

## Materials and Methods

We performed a secondary analysis of a retrospective cohort study on perioperative adverse events in women ≥ 61 years old undergoing prolapse surgery at a single academic center from January 2016 to May 2023. Only women undergoing MIS sacrocolpopexy by a board-certified urogynecologist (gynecology- or urology-trained reconstructive pelvic surgeons) were included. MIS sacrocolpopexies were done laparoscopically or robotically. We included patients who also underwent concurrent procedures including hysterectomy (supracervical and total) and incontinence surgery.

We defined older women as ≥ 70 years given the starting age of women in our cohort at the time of their MIS sacrocolpopexy was 61 years. In addition, studies in geriatric trauma, gastric cancer, orthopedic surgery, and thoracic surgery have identified 70 years as an age cutoff for increased risk of morbidity and mortality [19–22].

The primary outcome was a composite 8-week AE outcome which included intraoperative complications (occurring during the surgical procedure) and postoperative complications (up to 8 weeks after surgery). Intraoperative complications included conversion to laparotomy, bladder injury, ureteral injury, bowel injury, and estimated blood loss ≥ 500 mL. Postoperative complications included return to operating room, readmission, emergency room evaluation, discharge to skilled nursing facility, cardiac complication, venous thromboembolism, surgical site infections, wound dehiscence, blood transfusion, hematoma, ileus or small bowel obstruction, urinary tract infection, death, and other complications, defined as events that did not fall into the previously listed categories (e.g., vulvovaginal candidiasis, fall, *Clostridium difficile* infection, upper respiratory infection). Adverse events were identified on the basis of previously published literature examining intraoperative and postoperative outcomes in older surgical patients undergoing sacrocolpopexy [16, 17, 23, 24]. All data on perioperative and postoperative adverse events up to 8 weeks after MIS sacrocolpopexy were collected from the electronic medical record.

Secondary outcomes, including prolapse recurrence and mesh complication, were assessed using pelvic examinations performed by urogynecologists or general gynecologists, as documented through the most recent follow-up visit in the medical record. At the study’s institution, follow-up visits were performed at 4–6 weeks postoperatively and then as needed. Prolapse recurrence was defined as a symptomatic patient with at least stage 2 prolapse measured by Pelvic Organ Prolapse Quantification (POP-Q) score, an asymptomatic patient with prolapse leading edge at or distal to the hymen, or retreatment with pessary or surgery. Mesh complication included exposure or erosion of mesh. Patients with missing or incomplete postoperative examinations were excluded from the secondary analysis.

Demographic and clinical variables were compared between younger women (aged 61–69) and older women (aged ≥ 70) using *t*-test (or Mann–Whitney U) and chi-square (or Fisher’s exact) analyses. Multivariable logistic regression controlling for clinically relevant variables with *p* < 0.05 on univariate analyses were performed for the primary outcome as well as any individual AE with *p* < 0.20. Statistical analysis was conducted using STATA/SE 17.0 (StataCorp LLC, College Station, TX, USA). This study was granted IRB exemption (STUDY22070017).

## Results

A total of 709 women met criteria for the study. Within this cohort, 497 (70.1%) were aged 61–69, and 212 (29.9%) were aged ≥ 70 years (range 70–83 years). Table 1 displays demographic, clinical, and surgical characteristics of the cohort. There were no significant differences in BMI, smoking status, parity, race, or preoperative prolapse stage between younger and older women. Older patients were more likely to have hypertension (50.5% vs 38.6%, *p* < 0.01), diabetes (14.6% vs 8.7%, *p* = 0.02), a history of cancer (22.3% vs 11.9%, *p* < 0.01), and a higher Charlson Comorbidity Index (CCI) score (median 3 vs 2, *p* < 0.01). Rates of past prolapse surgery were similar between younger and older women, but older women were significantly less likely to have had a prior incontinence surgery (3.3% vs 8.1%, *p* = 0.02) and more likely to have had a prior hysterectomy (42.0% vs 32.0%, *p* = 0.01). Older patients had shorter length of surgery (164.8 ± 46.6 min vs 179.7 ± 49.0 min, *p* < 0.01) with lower estimated blood loss (median 35 mL vs 40 mL, *p* = 0.02). A robotic approach was used more often in surgeries for older women compared to younger women (52.4% vs 37.6%, *p* < 0.01).

**Table 1:** Demographic and clinical characteristics for sacrocolpopexy patients by age

	Age 61–69 years (*n* = 497)	Age ≥ 70 years (*n* = 212)	*p* value
General			
Age (years), *mean* ± *SD*	65.1 (2.6)	72.9 (2.5)	**< 0.01**
Body mass index (kg/m^2^), *mean* ± *SD*	27.2 (4.2)	26.9 (4.3)	0.41
Parity, *median* (*IQR*)	2 (2–3)	2 (2–3)	0.27
Current smoker,* n* (*%*)	19 (4.0)	5 (2.5)	0.49
Hispanic ethnicity,* n* (*%*)	2 (0.4)	0 (0)	1.00
Race			0.90
White,* n* (*%*)	469 (97.5)	201 (98.5)	
Black,* n* (*%*)	8 (1.7)	2 (1.0)	
Other,* n* (*%*)	4 (0.8)	1 (0.5)	
Preoperative pelvic organ prolapse quantification stage			0.96
Stage 2,* n* (*%*)	66 (13.3)	27 (12.7)	
Stage 3,* n* (*%*)	388 (78.2)	166 (78.3)	
Stage 4,* n* (*%*)	42 (8.5)	19 (9.0)	
Past medical history			
Hypertension,* n* (*%*)	182 (38.6)	104 (50.5)	**< 0.01**
Diabetes,* n* (*%*)	41 (8.7)	30 (14.6)	**0.02**
History of cancer,* n* (*%*)	56 (11.9)	46 (22.3)	**< 0.01**
Charlson Comorbidity Index, *median* (*IQR*)	2 (2–3)	3 (3–5)	**< 0.01**
Past surgical history			
Prior hysterectomy,* n* (*%*)	159 (32.0)	89 (42.0)	**0.01**
Prior prolapse surgery,* n* (*%*)	65 (13.1)	32 (15.1)	0.48
Prior incontinence surgery,* n* (*%*)	40 (8.1)	7 (3.3)	**0.02**
Surgical characteristics			
Robotic approach,* n* (*%*)	187 (37.6)	111 (52.4)	**< 0.01**
Concomitant hysterectomy,* n* (*%*)	337 (67.8)	123 (58.0)	**0.01**
Total hysterectomy, *n (%)*	79 (15.9)	37 (17.5)	0.61
Supracervical hysterectomy, *n (%)*	258 (51.9)	86 (40.6)	**< 0.01**
Concomitant incontinence procedure,* n* (*%*)	36 (7.2)	11 (5.2)	0.31
Length of surgery (minutes), *mean* ± *SD*	179.7 (49.0)	164.8 (46.6)	**< 0.01**
Estimated blood loss (mL), *median* (*IQR*)	40 (25–75)	35 (25–50)	**0.02**
Same Day Discharge,* n* (*%*)	424 (85.3)	188 (88.7)	0.23
Length of Follow-up (days), *median* (*IQR*)	227 (80–594)	289 (132–659)	0.11

*VTE* venous thromboembolism; *MI* myocardial infarction; *TIA* transient ischemic attack; *Hgb* hemoglobin

Missing data noted for the following variables: body mass index (*n* = 701), parity (*n* = 697), race (*n* = 685), Hispanic ethnicity (*n* = 667), current smoker status (*n* = 675), preoperative POP-Q stage (*n* = 708), past medical history including Charlson Comorbidity Index (*n* = 677), length of surgery (*n* = 670), estimated blood loss (*n* = 582)

Bolded values are significant (*p* < 0.05)

On univariate analysis, age ≥ 70 years was not significantly associated with 8-week perioperative AEs, as shown in Table 2 (17.0% vs 21.3%, *p* = 0.19). Particularly, AEs such as emergency room evaluations (2.8% vs 5.4%, *p* = 0.17), readmission (2.4% vs 2.6%, *p* = 1.00), postoperative UTI (6.2% vs 5.4%, *p* = 0.71), surgical site infections (1.9% vs 4.4%, *p* = 0.13), and ileus/small bowel obstruction (1.9% vs 0.8%, *p* = 0.25) were infrequent and similar between older and younger women. Older women did not experience any intraoperative complications besides bladder injury (1.9%). Older women and younger women had similar lengths of follow-up (median 289 days vs 227 days, *p* = 0.11, Table 1) and did not differ in rates of prolapse recurrence (10.9% vs 11.1%, *p* = 0.93) and mesh complication (1.9% vs 1.2%, *p* = 0.50).

**Table 2:** Adverse events in sacrocolpopexy patients by age

	Age 61-69 years (*n*=497)	Age ≥ 70 years (*n*=212)	*p* value
Composite adverse events	106 (21.3)	36 (17.0)	0.19
Intraoperative complications, *n* (*%*)			
Conversion to laparotomy	0 (0)	0 (0)	
Bladder injury	5 (1.0)	4 (1.9)	0.46
Ureteral injury	2 (0.4)	0 (0)	1.00
Bowel injury	3 (0.6)	0 (0)	0.56
EBL ≥ 500	2 (0.5)	0 (0)	1.00
Postoperative adverse events within 8 weeks, *n* (*%*)			
Return to operating room	4 (0.8)	1 (0.5)	1.00
Readmission	13 (2.6)	5 (2.4)	1.00
ED evaluation	27 (5.4)	6 (2.8)	0.17
Discharge to SNF	0 (0)	0 (0)	
Cardiac complication	3 (0.6)	1 (0.5)	1.00
Venous thromboembolism	1 (0.2)	1 (0.5)	0.51
Transfusion	0 (0)	1 (0.5)	0.30
Hematoma	3 (0.6)	0 (0)	0.56
Wound dehiscence	3 (0.6)	1 (0.5)	1.00
Ileus/small bowel obstruction	4 (0.8)	4 (1.9)	0.25
Postoperative UTI	27 (5.4)	13 (6.2)	0.71
Death	0 (0)	1 (0.5)	0.30
Other complications*	32 (6.6)	10 (4.9)	0.38
Surgical site infection within 8 weeks, *n* (*%*)	22 (4.4)	4 (1.9)	0.13
Superficial SSI	20 (4.0)	4 (1.9)	0.18
Deep SSI	0 (0)	0 (0)	
Organ-space SSI	2 (0.4)	0 (0)	1.00
Long-term Complications, *n* (*%*)			
Prolapse Recurrence	55 (11.1)	23 (10.9)	0.93
Mesh Complication	6 (1.2)	4 (1.9)	0.50

*ED*, emergency department; *SNF*, skilled nursing facility, *UTI*, urinary tract infection; *EBL*, estimated blood loss, *SSI*, surgical site infection

All adverse events and surgical site infections occurred within 8 weeks postoperative except for mesh complication and prolapse recurrence which could occur at any point during follow-up

**Composite adverse events** outcome includes all 8-week adverse events: intraoperative complications (conversion to open, bladder injury, bowel injury, ureteral injury, EBL ≥ 500), venous thromboembolism, surgical site infections (superficial, deep, organ), wound dehiscence, blood transfusion, hematoma, ileus or small bowel obstruction, return to operating room, emergency room evaluation, non-routine admission, re-admission, postoperative urinary tract infection, and other complications

*Other complications included skin rash/hives, ankle sprain, fallopian tube cancer, endometrial cancer, inferior epigastric artery injury, tooth abscess, vulvovaginal candidiasis, hyperglycemia, fall, vertigo, upper respiratory infection, bronchitis, influenza, COVID, C. difficile infection, pyelonephritis, lower extremity neuropathy, acute kidney injury, Crohn’s flare, motorcycle accident

Bolded values are significant (*p* < 0.05)

The composite AE outcome was not associated with age ≥ 70 years on our multivariable logistic regression model that controlled for CCI score, robotic approach, and concomitant hysterectomy (adjusted odds ratio (aOR) 0.64, 95% confidence interval (CI) [0.38–1.07], Table 3). Women aged ≥ 70 years had a 73% lesser adjusted odds of surgical site infections on multivariable analysis (95% CI [0.08–0.93]).

**Table 3:** Univariate and multivariable analysis of impact of age on adverse events in older sacrocolpopexy patients

	Age ≥ 70 years Unadjusted OR [95% CI]	*p* value	Age ≥ 70 years Adjusted OR* [95% CI]	*p* value
Composite adverse events	0.75 [0.50–1.15]	0.19	0.64 [0.38–1.07]	0.09
ED evaluation	0.51 [0.21–1.25]	0.14	0.37 [0.13–1.04]	0.06
Surgical site infection (all)	0.42 [0.14–1.22]	0.11	0.27 [0.08–0.93]	**0.04**

*OR* odds ratio; *CI* confidence interval

^*^Multivariable analyses controlled for Charlson Comorbidity Index score, robotic approach, and concomitant hysterectomy

Bolded values are significant (*p* < 0.05)

## Discussion

In this select cohort of older women undergoing MIS sacrocolpopexy, we did not find a difference in odds of perioperative AEs within 8 weeks, prolapse recurrence, or mesh complication between women aged 61–69 and ≥ 70 years. Controlling for CCI score, robotic approach, and concomitant hysterectomy, older women had lower odds of surgical site infection compared to younger women, although the overall rate of surgical site infection was low in both groups.

Our study shows that women age ≥ 70 years undergoing MIS sacrocolpopexy do not have an increased risk of perioperative AEs compared to their younger counterparts. A study in 2020 by Turner et al. found an increased risk of major complications in women age ≥ 65 with similar comorbidities (hypertension, diabetes, and/or stroke) undergoing MIS sacrocolpopexy [14]. Their study used older data from 2009–2012, and the MIS sacrocolpopexies included in their study had a longer average operating time (about 250 min) and length of hospital stay (1.4 days) than our cohort. Additionally, only 20.6% of their older patient cohort underwent robotic-assisted sacrocolpopexy compared to 52.6% in our study. Hong et al. examined only robotic-assisted sacrocolpopexy among 100 women over age ≥ 65 and found no significant difference in overall rate of perioperative adverse events compared to 227 women < 65 years [17]. Chaudhry et al. utilized the National Surgical Quality Improvement Program database from 2010–2013 to examine MIS sacrocolpopexy and found that age, when stratified into < 60, 60–64, 65–69 70–74, and 75+ years, was not associated with an increased odds of perioperative AEs [16]. Similarly, Gluck et al. reported no increased risk in 30-day perioperative complications in patients aged 75 and older undergoing laparoscopic sacrohysteropexy and sacrocolopoexy, compared to patients < 65 years and 65–75 years [15]. Lastly, among 440 patients who underwent MIS sacrocolpopexy at a single institution from 2006–2016, Edge et al. found no difference in 6-week postoperative complications between patients ≥ 65 years and 55–64 years [13]. Compared to these studies, we used a higher age cutoff for “older” (≥ 70 years compared to ≥ 65 years) and our comparison group was greater in age (aged 61–69 years). We examined a comprehensive set of AEs up to 8 weeks postoperative compared to prior studies which describe AEs up to 4–6 weeks postoperative. This provides stronger evidence that there does not appear to be an elevated risk of adverse events after MIS sacrocolpopexy among women in this older age bracket.

Despite having greater medical comorbidities including higher CCI score, hypertension, diabetes, and history of cancer, the older women in our cohort sustained low rates of AEs after MIS sacrocolpopexy. Our results reiterate those of other studies which have found low rates of perioperative AEs such as pelvic organ injury, bowel obstruction, postoperative UTIs, readmissions, and reoperations in women undergoing MIS urogynecologic surgery [12–15, 17, 23, 24]. It is important to note that prolapse surgery, which is done for quality-of-life enhancement, allows for optimization in the selection of surgical candidates and decision for surgical approach. Given this, the women in our cohort are not representative of the general population, which may explain the low rates of perioperative AEs in comparison to other surgical specialties.

In our data, women aged ≥ 70 years did not have an elevated risk of long-term mesh complication or prolapse recurrence compared to younger women aged 61–69 years. Rates of prolapse recurrence and mesh exposure in the subset of older patients were low (10.9% and 1.9%, respectively). In a study looking at long-term prolapse recurrence and mesh exposure by Thomas et al., 11.6% of patients undergoing all modalities of sacrocolpopexy (open, laparoscopic, robotic) experienced prolapse recurrence on examination (stage 2 or greater) with a median follow-up time of 0.5 years [21]. In the same study, 5.2% of patients had mesh and/or suture exposure on examination. Lavelle et al. reported prolapse recurrence among 47/633 (7.4%) women undergoing MIS sacrocolpopexy with a median follow-up time of 47 weeks [25]. Our study supports the notion that rates of prolapse recurrence and mesh exposure in women ≥ 70 undergoing MIS sacrocolpopexy are comparable to rates found in the general population of women having this procedure who have a similar length of follow-up. However, the interpretation of this data is limited due to the retrospective nature of our study and the possibility that patients with recurrence or mesh complication sought care at other institutions. Additionally, some studies have shown that the risk of prolapse recurrence and mesh exposure increases with length of follow-up through multiple years [26, 27]. Most of the patients in our study did not have follow-up visits beyond a year which limited our ability to assess for prolapse recurrence or mesh exposure at later time points. These findings highlight the need for longer-term follow-up in older patients to accurately detect delayed complications following MIS sacrocolpopexy.

Strengths of our study include our relatively larger cohort of older women undergoing MIS sacrocolpopexy compared to previously published studies and the longer length of AE follow-up. Additionally, we examined other outcomes of interest such as prolapse recurrence and mesh complication. Our study cohort is limited by its retrospective design at a single institution of a predominantly White population, which limits the generalizability of our findings. Furthermore, although we looked at AEs within 8 weeks, it would be beneficial to look at longer term AEs (i.e., 3 months and 1 year) among an older population.

In conclusion, our study provides further reassuring data that MIS sacrocolpopexy is safe and effective in older women.

## Data Availability

The data that support the findings of this study are available from the corresponding author, SWZ, upon reasonable request.
